# Predicting gene targets from integrative analyses of summary data from GWAS and eQTL studies for 28 human complex traits

**DOI:** 10.1186/s13073-016-0338-4

**Published:** 2016-08-09

**Authors:** Jennifer M. Whitehead Pavlides, Zhihong Zhu, Jacob Gratten, Allan F. McRae, Naomi R. Wray, Jian Yang

**Affiliations:** Queensland Brain Institute, University of Queensland, Brisbane, Queensland Australia

**Keywords:** Genome-wide association studies (GWAS), Expression quantitative trait loci (eQTL), Summary data-based Mendelian randomization (SMR), Complex traits

## Abstract

Genome-wide association studies (GWAS) have identified hundreds of genetic variants associated with complex traits and diseases. However, elucidating the causal genes underlying GWAS hits remains challenging. We applied the summary data-based Mendelian randomization (SMR) method to 28 GWAS summary datasets to identify genes whose expression levels were associated with traits and diseases due to pleiotropy or causality (the expression level of a gene and the trait are affected by the same causal variant at a locus). We identified 71 genes, of which 17 are novel associations (no GWAS hit within 1 Mb distance of the genes). We integrated all the results in an online database (http://www.cnsgenomics/shiny/SMRdb/), providing important resources to prioritize genes for further follow-up, for example in functional studies.

**Electronic supplementary material:**

The online version of this article (doi:10.1186/s13073-016-0338-4) contains supplementary material, which is available to authorized users.

## Background

Genome-wide association studies (GWAS) have identified thousands of genetic loci associated with various complex traits, disorders, and diseases [1, 2]. The GWAS paradigm exploits the linkage disequilibrium (LD) correlation structure of the genome, which means that the majority of the variation in the genome can be captured in a cost-effective way by genotyping only a few hundred thousand variants, followed by imputation of non-genotyped variants using a densely genotyped reference panel [3]. However, the LD structure also means that identified associations frequently point to genomic regions that harbor many genes, and it is extremely difficult to prioritize among these genes to identify the most functionally relevant genes using GWAS data alone. Laboratory-based follow-up of the associated regions is costly and prohibitive given the number of putatively causal variants in a typical genome-wide significant locus. GWAS of gene expression levels has allowed identification of expression quantitative trait loci (eQTL) [4–6]. Several recent methods [7–11] have used analytical approaches to integrate eQTL and complex trait associations as strategies to prioritize genes for further studies. In this study, we apply the recently developed summary data-based Mendelian randomization (SMR) method to 28 complex traits (including diseases), which have GWAS summary statistics available in the public domain, to obtain a list of genes to prioritize for further follow-up such as functional studies, and develop a database to query all the data and results. We use the SMR method because: it implements a transcriptome-wide association analysis in a formal statistical framework using summary data so that the statistical power is increased by using the latest GWAS and eQTL data of very large sample size; it provides a test to distinguish pleiotropy (or causality) from linkage (see below for more details) [11]; and it is implemented in a user-friendly software tool [12, 13].

## Construction and content

Details of the SMR method can be found in the Zhu et al. paper [11]. In brief, SMR applies the principles of Mendelian randomization (MR) to jointly analyze GWAS and eQTL summary statistics in order to test for association between gene expression and trait due to a shared causal variant at a locus. Mendelian randomization is an instrumental variable analysis approach that uses genetic variant(s) as instrumental variable(s) (Z) to test whether an exposure (X) has a causal effect on an outcome (Y) [14, 15]. Equivalently, it is an analysis to test whether the effect of Z on Y is mediated by X (a model of Z - > X - > Y). The instrumental variable estimate of the effect of X on Y (*b*_XY_) can be expressed as *b*_XY_ = *b*_ZY_/*b*_ZX_, where *b*_ZY_ is the effect size of Z on Y and *b*_ZX_ is the effect size of Z on X [16]. This approach is usually used to test for the causative effect of a modifiable risk factor on health outcomes but the same principle can be used to test whether the effect size of a SNP (Z) on a trait (Y) identified from GWAS is mediated by the expression level of a gene (X). The SMR test [11] is a two-sample MR approach [17, 18]. It allows us to estimate and test *b*_XY_ using summary data from independent studies [11]. For the purpose of testing for the association between gene expression and trait, it uses the estimate of SNP effect on the trait (*b*_ZY_) from GWAS summary data and the estimate of SNP effect on gene expression (*b*_ZX_) from summary data of an independent eQTL study. In this case, trait is the outcome (Y), gene expression is the exposure (X), and the top cis-eQTL that is strongly associated with gene expression is used as the instrument (Z) (we used cis-eQTL with *P*_eQTL_ <5e-8 in this study). Here we use “association” rather than “causal association” because previous results [11] suggest that there are at least three models consistent with a significant association from the SMR test using only a single genetic variant. These models are causality (Z - > X - > Y), pleiotropy (Z - > X and Z - > Y), and linkage (Z_1_ - > X, Z_2_ - > Y, and Z_1_ and Z_2_ are in LD). We provide details below of a test to distinguish pleiotropy (or causality) from linkage that is of less biological interest. The purpose of this study is to identify genes whose expression levels are associated with complex traits due to a shared causal variant. We therefore do not further distinguish between causality and pleiotropy (which is also impossible to achieve using only the cis-eQTLs).

As mentioned above, significant SMR results could also reflect linkage (i.e. the top associated cis-eQTL being in LD with two distinct causal variants, one affecting gene expression and the other affecting trait variation), which may be of less interest, at least in the first round of gene prioritization. To exclude SMR results that may reflect linkage, Zhu et al. [11] proposed the heterogeneity in dependent instruments (HEIDI) test, which considers the pattern of associations using all the SNPs that are significantly associated with gene expression (eQTLs) in the cis-region. The null hypothesis is that there is a single causal variant affecting trait and gene expression (pleiotropy or causality), which is of biological interest and should be prioritized for follow-up studies. The alternative hypothesis is that gene expression and trait are affected by two distinct causal variants, which is of less biological interest. Under the null hypothesis that there is a single causal variant, *b*_XY_ estimated at any of the cis-SNPs that are associated with gene expression (e.g. SNPs with *P*_eQTL_ <1.6 × 10^−3^, equivalent to *χ*^2^ > 10) is expected to be equal to that estimated at the top associated cis-eQTL (see Equation 7 of Zhu et al. [11] for more details). Therefore, it is equivalent to test whether there is heterogeneity in *b*_XY_ estimated at the significant cis-eQTLs (null hypothesis: no heterogeneity, causality or pleiotropy model; alternative hypothesis: heterogeneity, linkage model). Note that the HEIDI test takes into account non-independence of cis-eQTLs due to LD (using individual-level data from a reference sample to estimate LD between the cis-SNPs). Probes that show evidence of heterogeneity (e.g. *P*_HEIDI_ <0.05) are rejected.

The previous SMR study analyzed three traits (body mass index (BMI), height, and waist-to-hip ratio adjusted by BMI) and two diseases (rheumatoid arthritis and schizophrenia) and identified 21 novel genes (genes that passed the SMR and HEIDI tests and that are located >1 Mb from the nearest GWAS hit) [11]. In this study, the SMR analysis is extended to an additional 28 complex traits and diseases (Table 1) which have summary data available in the public domain from large-scale GWAS. The results from the SMR analyses are made available in an online query database (http://www.cnsgenomics.com/shiny/SMRdb/) [13], which is implemented in R Shiny.

**Table 1:** GWAS information and SMR results for 28 complex traits and diseases

Trait/Disease	N for quantitative traits or N_cases_/N_controls_	Number of genes (probes) GWS for the SMR test	Number of genes (probes) not rejected by the HEIDI test	Reference
Attention deficit and hyperactivity disorder (ADHD)	2787/2635	–	–	[22]
Alzheimer's disease (ALZ)	17,008/37,154	7 (8)	2 (2)	[23]
Autism spectrum disorder (ASD)	13,088/16,664	–	–	[24]
Bipolar disorder (BIP1)	7481/9250	1 (1)	1 (1)	[25]
Major depressive disorder (MDD)	9240/9519	–	–	[26]
Inflammatory bowel disease (IBD)	12,882/21,770	37 (40)	14 (14)	[19]
Crohn's disease (CD)	5956/14,927	29 (33)	11 (12)	[19]
Ulcerative colitis (UC)	6968/20,464	17 (17)	6 (6)	[19]
Coronary artery disease (CAD)	60,801/123,504	9 (9)	5 (5)	[27]
Diastolic blood pressure (DBP)	69,395	5 (5)	–	[28]
Systolic blood pressure (SBP)	69,395	4 (4)	–	[28]
High-density lipoproteins (HDL)	93,561	38 (43)	12 (13)	[29]
Low-density lipoproteins (LDL)	89,138	28 (31)	6 (7)	[29]
Total cholesterol (TC)	93,845	40 (43)	8 (9)	[29]
Triglycerides (TG)	90,263	22 (25)	2 (2)	[29]
Type-2 diabetes (T2D)	12,171/56,862	–	–	[30]
Fasting glucose (FGLUCOSE)	38,422	4 (5)	–	[31]
Fasting insulin (FINSULIN)	23,823	–	–	[31]
Cigarettes per day (CIGPERDAY)	38,181	2 (3)	1 (2)	[32]
Ever smoked (EVERSMOKED)	74,035	–	–	[32]
College completion (COLLEGE) [33]	95,427	1 (1)	1 (1)	[33]
Education attainment (EDUYEARS)	101,069	3 (3)	3 (3)	[33]
Intelligence quotient (IQ)	17,989	–	–	[34]
Agreeableness (AGREE)	17,375	–	–	[35]
Conscientiousness (CONS)	17,375	–	–	[35]
Extraversion (EXTRAVERT)	17,375	–	–	[35]
Neuroticism (NEUROTIC)	63,661	–	–	[36, 37]
Openness (OPEN)	17,375	–	–	[35]
Total		247 (271)	71 (77)	

Probe: a specific DNA sequence designed on a gene expression array to capture a transcript

## Utility and discussion

After quality control (QC) steps [11], associations between 5967 probes and 757,479 SNPs from the blood gene expression study by Westra et al. [5] were used in the analysis. The Westra eQTL summary data are available in the public domain and on the SMR website [12]. It should be noted that all the probes included in the analysis have at least a cis-eQTL at *P*_eQTL_ <5 × 10^–8^. For each probe, the top associated cis-eQTL was used as the instrument for the SMR test. The SMR test was performed for each of the 5967 probes on 28 traits and disorders/diseases (Additional file 1: Table S1). The genome-wide significance level for the SMR test, corrected for multiple testing, is defined as 0.05/5967 = 8.4 × 10^–6^. For probes with *P*_SMR_ <8.4 × 10^–6^, we conducted the HEIDI test and retained for further investigation only those probes with little evidence of heterogeneity *P*_HEIDI_ ≥0.05. All the analyses were performed using the SMR software tool [11, 12]. We particularly emphasized results that are considered to be novel, i.e. no previously identified SNP, reported as genome-wide significant in the primary GWAS paper, within a 1 Mb window of the probes. We identified 247 gene-trait associations (271 probes) with *P*_SMR_ <8.4 × 10^–6^ (Additional file 1: Table S2). After application of the HEIDI test (*P*_HEIDI_ ≥0.05), this was reduced to 71 gene-trait associations (77 probes) (Additional file 1: Table S3). Of these, 17 gene-trait associations were considered novel (Table 2 and Additional file 1: Table S4).

**Table 2:** Seventeen novel genes identified in the SMR Analysis. Novel genes are genes that have passed both the SMR and HEIDI tests (*P*_SMR_ <8.4E-06 and *P*_HEIDI_ ≥ 005), have not previously been identified as GWS, and no GWS loci within 1 Mb window reported in the primary GWAS paper (full results are given in Additional file 1: Table S4)

Trait	Probe ID	Gene	Top cis-eQTL	Allele Freq	*P*_eQTL_	*P*_GWAS_	*P*_SMR_	*P*_HEIDI_	nsnp
BIP1	ILMN_1665280	*SPCS1*	rs998909	0.420	2.1E-39	6.8E-07	3.4E-06	0.15	155
CAD	ILMN_1713380	*EIF2B2*	rs175016	0.475	1.8E-278	4.7E-06	5.6E-06	0.23	189
	ILMN_1712430	*ATP5G1*	rs1962412	0.281	1.3E-44	7.4E-07	3.0E-06	0.27	127
CD	ILMN_1718852	*PLCL1*	rs2117339	0.486	6.7E-30	8.0E-07	6.0E-06	0.14	216
	ILMN_2122952	*CISD1*	rs1199098	0.214	<1.0E-300	1.5E-06	1.7E-06	0.17	241
	ILMN_2122953		rs1550773	0.212	<1.0E-300	2.0E-06	2.2E-06	0.13	217
COLLEGE	ILMN_1723684	*DARC*	rs12075	0.456	4.8E-107	3.3E-06	5.4E-06	0.47	110
EDUYEARS	ILMN_1718023	*APEH*	rs3197999	0.291	1.1E-27	5.7E-07	5.5E-06	0.08	88
	ILMN_2343048	*ABCB9*	rs1615350	0.248	9.1E-43	2.0E-06	7.2E-06	0.75	53
	ILMN_1738369	*TUFM*	rs8049439	0.405	<1.0E-300	1.5E-07	1.7E-07	0.11	37
HDL	ILMN_1684227	*GPR146*	rs1997243	0.155	2.2E-300	2.4E-07	3.1E-07	0.22	130
IBD	ILMN_1697409	*TNFRSF14*	rs734999	0.483	2.1E-90	2.3E-07	5.4E-07	0.98	64
	ILMN_1727709	*GPBAR1*	rs2292550	0.405	8.3E-43	6.3E-08	4.9E-07	0.24	109
	ILMN_1684628	*ZFP90*	rs1182968	0.219	<1.0E-300	3.3E-06	3.6E-06	0.90	311
LDL	ILMN_1718706	*ERAL1*	rs901975	0.202	6.5E-46	2.2E-06	6.9E-06	0.19	66
UC	ILMN_1744713	*PARK7*	rs3766606	0.173	1.1E-53	5.7E-08	3.0E-07	0.09	195
	ILMN_1727709	*GPBAR1*	rs2292550	0.405	8.3E-43	1.2E-07	8.1E-07	0.12	109
	ILMN_1683811	TNPO3	rs3807306	0.496	1.4E-150	2.3E-06	3.3E-06	0.69	125

*P*_*eQTL*_ *p* value of the top associated cis-eQTL of the probe, *P*_GWAS_ GWAS *p* value of the top cis-eQTL, *P*_SMR_ *p* value for gene-trait association from the SMR test, *P*_HEIDI_ *p* value from HEIDI test to indicate whether the gene-trait association is due to a single shared genetic variant (the smaller *P*_HEIDI_ the more likely that there are more than one genetic variant)

There were 15 genes associated with more than one trait or disease (Additional file 1: Table S5). Where a gene was associated across more than one trait, there was a strong correlation between the traits, with only two cross trait associations being between disparate traits or diseases. Crohn’s disease (CD) and ulcerative colitis (UC) are chronic gastrointestinal disorders that represent as intestinal inflammation; collectively they are known as inflammatory bowel disease (IBD). GWAS to date have identified 200 loci associated with IBD [19], 71 with CD [20], and 47 with UC [21], as well as evidence for trans-ancestry shared genetic risk for IBD [19]. The SMR analyses predicted ten gene targets for a combination of IBD, CD, and UC (Additional file 1: Table S6), of which four were novel gene associations (in total there were two novel gene associations for CD and three each for IBD and UC). The other traits that shared gene associations were the lipids, i.e. high-density lipoprotein (HDL), low-density lipoprotein (LDL), and total cholesterol (TC) (Additional file 1: Table S7).

The results from this analysis can be queried and viewed in the online application [13]. Results from the initial Zhu et al. study are also included in this database. We intend that as more GWAS summary data becomes available, SMR analysis will be conducted using the summary data and the results database will be updated accordingly. This application enables users to query the database by trait, gene, or both and apply thresholds based on the *p* value from the SMR method and the HEIDI test. In addition, Manhattan plots are given based on the *p* value from the SMR analysis and regional association plots are provided for those probe-trait associations that pass both the SMR and HEIDI tests.

## Conclusion

SMR, as indicated by the results, provides a means of using summary statistics from GWAS and eQTL data to prioritize likely functionally relevant genes within previously identified regions of association and in some cases identify novel gene associations.

## Abbreviations

CD, Crohn’s disease; eQTL, Expression quantitative trait loci; GWAS, Genome-wide association study; HDL, High-density lipoprotein; HEIDI, Heterogeneity in dependent instruments; IBD, Inflammatory bowel disease; LD, Linkage disequilibrium; LDL, Low-density lipoprotein; MR, Mendelian randomization; QC, Quality control; SMR, Summary data-based Mendelian randomization; TC, Total cholesterol; UC, Ulcerative colitis

## Additional files

Additional file 1: Table S1. GWAS information. **Table S2.** SMR results (*P*_SMR_ <8.4 × 10^–6^). **Table S3.** SMR and HEIDI results (*P*_HEIDI_ ≥0.05). **Table S4.** Novel genes. **Table S5.** Genes across more than one trait. **Table S6.** IBD, CD, and UC gene associations. **Table S7.** HDL, LDL, and TC gene associations. (XLSX 141 kb) Additional file 2: Full list of acknowledgements. (DOCX 109 kb)
