# Supplementary material for: Predicting gene targets from integrative analyses of summary data from GWAS and eQTL studies for 28 human complex traits
Source: Genome Med. 2016 Aug 9;8:84. doi: 10.1186/s13073-016-0338-4 (PMC4979185; doi:10.1186/s13073-016-0338-4)
Supplement: Additional file 2: — Full list of acknowledgements. (DOCX 109 kb) [file 13073_2016_338_MOESM2_ESM.docx]

**Text S1: Acknowledgements**

Psychiatric Genomics Consortium for data on Bipolar Disorder, Autism Spectrum Disorder, Attention Deficit and Hyperactivity Disorder, and Major Depressive Disorder; the CARDIoGRAMplusC4D investigators for data on coronary artery disease / myocardial infarction; the International Consortia for Blood Pressure for data on diastolic and systolic blood pressure; the MAGIC investigators for glycaemic traits; the DIAbetes Genetics Replication And Meta-analysis group for data on Type 2 Diabetes; the Global Lipids Genetics Consortium for data on lipids, total cholesterol and triglycerides; the Tobacco and Genetics Consortium for data on smoking behaviour; the IBD genetics group for data on Inflammatory Bowel Disease, Crohn’s disease and ulcerative colitis; the Social Sciences Genetic Association Consortium for data on educational attainment and IQ; the Genetics of Personality Consortium for data on five personality traits; and the International Genomics of Alzheimer's Project (IGAP) for providing summary results data for these analyses. The investigators within IGAP contributed to the design and implementation of IGAP and/or provided data but did not participate in analysis or writing of this report. IGAP was made possible by the generous participation of the control subjects, the patients, and their families. The i–Select chips was funded by the French National Foundation on Alzheimer's disease and related disorders. EADI was supported by the LABEX (laboratory of excellence program investment for the future) DISTALZ grant, Inserm, Institut Pasteur de Lille, Université de Lille 2 and the Lille University Hospital. GERAD was supported by the Medical Research Council (Grant n° 503480), Alzheimer's Research UK (Grant n° 503176), the Wellcome Trust (Grant n° 082604/2/07/Z) and German Federal Ministry of Education and Research (BMBF): Competence Network Dementia (CND) grant n° 01GI0102, 01GI0711, 01GI0420. CHARGE was partly supported by the NIH/NIA grant R01 AG033193 and the NIA AG081220 and AGES contract N01–AG–12100, the NHLBI grant R01 HL105756, the Icelandic Heart Association, and the Erasmus Medical Center and Erasmus University. ADGC was supported by the NIH/NIA grants: U01 AG032984, U24 AG021886, U01 AG016976, and the Alzheimer's Association grant ADGC–10–196728.
